# The impact of age-related changes in the skull on sex estimation using morphoscopic traits

**DOI:** 10.1007/s00414-025-03568-1

**Published:** 2025-08-06

**Authors:** Sarah-Kelly Houston, Desiré Brits, Jolandie Myburgh, Leandi Liebenberg

**Affiliations:** 1 Department of Anatomy, University of Pretoria, Pretoria, South Africa; 2 Human Variation and Identification Research Unit, School of Anatomical Sciences, University of the Witwatersrand, Johannesburg, South Africa

**Keywords:** Forensic anthropology, Morphoscopic, Aging, Sexual dimorphism, Walker method, South Africa

## Abstract

The five sexually dimorphic traits of the skull described by Walker are frequently employed in skeletal analyses for sex estimation. Previous research has highlighted various changes in the craniofacial complex associated with advancing age, as cranial remodelling persists into adulthood. Age has been recognised as one potential factor contributing to misclassification when using the Walker traits. This study aimed to evaluate the influence of age on the expression of sexually dimorphic cranial traits and the impact of that influence on sex estimation in a South African sample. The traits were scored on a sample of 453 skulls between 14 and 108 years of age with an equal sex distribution. Only the nuchal crest exhibited significant differences in score distributions among the females in the sample, indicating differences between individuals younger than 40 years and those older than 40 years. Classification models showed a slight increase in accuracy for younger individuals when age-specific standards were applied, while accuracy for older individuals decreased slightly. However, the difference in accuracy with age-specific standards was comparable to accuracies obtained with all ages pooled. The results suggest age did not have as substantial an impact on trait expression as population affinity, and prior knowledge of age did not significantly influence the accuracy of the method. Thus, the pre-selection of age before the estimation of sex using the Walker traits is not currently necessary when conducting skeletal analyses in a contemporary South African population. However, the use of population-specific standards is paramount to more accurate classification.

**Supplementary Information:**

The online version contains supplementary material available at 10.1007/s00414-025-03568-1.

## Introduction

Sexual dimorphism, referring to the size and shape differences between biological males and females, has been observed throughout the human skeleton [1, 2]. Various osteometric and morphoscopic techniques exist to quantify sexual dimorphism. Among these methods the Walker [3] method is a widely accepted technique to estimate sex when the skull is available for analyses. The Walker [3] method involves the examination of five morphoscopic traits on the cranium and mandible, namely the glabella, supra-orbital margins, nuchal crest, mastoid processes, and mental eminence. Each trait is scored on an ordinal scale, ranging between 1 (the maximum female-leaning expression) and 5 (the maximum male-leaning expression) [3]. The methodology has been validated through numerous studies worldwide, with several authors noting population-specific variations among the traits [4–6]. Notably, the South African population has been shown to exhibit differences (such as decreased sexual dimorphism) compared to the North American reference sample used in the development of the method [4]. Population differences have also been reported for most of the traits among different population groups within South Africa, with varying degrees of overlap and trait score frequencies for black, coloured, white and Indian South Africans [4, 7].

In addition to population affinity, age has been recognised as a significant factor influencing sexual dimorphism, potentially impacting the accuracy of sex estimation [8, 9]. Throughout adulthood factors such as fluctuations in hormone levels, mechanical stress, remodelling, and a decline in metabolic activity and osteoblast production can contribute to changes in the skeleton [10]. In particular, size and morphological changes in the craniofacial skeleton, which results from developmental processes, degenerative processes or a combination of both, have been extensively documented in the literature [9, 11–17]. Albert and colleagues [9] report craniofacial aging (including the increase in the size and change in shape of the cranium) likely related to maturation continuing until at least the third decade of life; however, additional modifications more closely related to degeneration may persist into the fifth and sixth decades.

Degenerative age-related changes in the skull typically manifest in the form of tooth loss leading to alveolar bone resorption, rougher granulation at muscle attachment sites, structural alterations in the vault due to weakening of the masticatory muscles, as well as differential thickening or thinning of the cranial bones [12]. In craniometric studies, transverse enlargement was reported between the third and eighth decades of life, with most craniofacial dimensions increasing in small increments [9]. Moreover, anterior facial height, as well as mandibular length, was shown to also increase with advancing chronological age [4, 8, 9]. While differences are frequently observed when comparing the crania of young and old adults, some differences have also been noted among younger groups. More specifically, Ross and Williams [11] found significant shape differences among native African individuals aged between 20 and 25 years. Significant size differences were also identified around the 25-year mark [11]. These changes in younger adults are more developmental in nature and have been attributed to an increase in the size of the paranasal sinuses, eruption of the third molars and/or alveolar remodelling [11].

Dentition, whether still developing or undergoing antemortem loss, plays a significant role in facial morphology [11]. Albert et al. [9]. report a substantial increase in lower anterior facial height with the extrusion of the lower incisors, with the most pronounced increase occurring in the early twenties. Similarly, Small et al. [18]. found that tooth loss and edentulism influence the maxillary and mandibular alveolar bone, altering upper facial height and palatal shape. Mandibular alveolar resorption following antemortem tooth loss also contributes to a reduction in the prominence of the mental eminence. However, edentulism affects more than just the facial skeleton. In a sample of edentulous South Africans, anterior and inferior flexion of the basicranium was observed, leading to inferior displacement of the external occipital protuberance (nuchal crest) and anterior-inferior movement of the mastoid processes when compared to individuals with intact dentition [18]. It is important to acknowledge that tooth loss is multifactorial and not solely age-related, as socio-economic status and poverty have also been identified as contributing factors [19].

Biological age and sexual dimorphism are closely linked, as the degree of sexual dimorphism in a skeletal feature depends on its growth rate. More specifically, features that develop earlier tend to exhibit less sexual dimorphism compared to those that mature later [20, 21]. In the cranium, sexual dimorphism (both in size and shape) primarily results from differential growth patterns of its various functional matrices [17, 20]. For instance, the basicranium matures earlier than the facial skeleton, often leading to lower expression of sexual dimorphism in the cranial base. Regarding the cranial traits used in the Walker [3] method, the nuchal crest is the first to reach maturity, while the mastoid process is among the last [21]. Despite these differences in maturation, full expression of sexual dimorphism in these traits has been observed during adolescence [20], allowing for sex estimation in adolescent individuals. Additionally, growth rates, or ontogenetic trajectories, vary not only among different cranial regions but also among individuals. Females typically enter puberty earlier than males and reach their adult skeletal state sooner, whereas males experience an extended growth period, resulting in larger and more robust cranial features [20].

The majority of previous studies have assessed age-related changes using geometric morphometric techniques or standard cranial measurements [9, 11, 13–15, 18]. But few studies have discussed the practical implications of aging on morphoscopic techniques applied to the skull [22]. Walker [23] indicated that cranial features associated with sex may change in their degree of expression throughout adult life. Most of these changes are observed among males with a shift from gracile (lower trait scores) to more robust (higher trait scores) with increasing age; as such, males younger than 30 years of age have been noted to exhibit more female-leaning cranial features [23, 24]. Female crania retain fairly gracile features throughout puberty and into young adulthood, with changes occurring in older females. More specifically, post-menopausal females tend to exhibit more male-leaning features [23] and it has been suggested that sex estimation using the cranium should not be attempted in females older than 55 years of age, as misclassification is likely to occur [23, 24]. More recent North American studies have indicated that a weak correlation exists between age and the Walker [3] traits, but that age did not have a large impact on expression of the traits [10, 16, 22]. However, further research is required for different population groups as the extent of age-related changes to bone, as well as sexual dimorphism, may differ between populations (possibly due to differences in genetics, diet and environment). Thus, it is essential to assess if the influence of age-related changes on the degree of expression of sexually dimorphic cranial traits vary between populations.

The South African-specific standards created by Krüger et al. [4] based on the five Walker [3] traits made use of the Pretoria Bone Collection (PBC). As is the case with numerous skeletal collections across the globe, many of the individuals that are accessioned often tend be of more advanced ages [26–28]. Notably, the sample used by Krüger et al. [4]. demonstrates a disparity between the groups, with the mean age for the white South Africans being nearly 20 years older than the black South Africans (68 years versus 50 years, respectively). Thus, if age impacts the expression of sexual dimorphism it could lead to greater misclassifications for one group if the same standards are applied regardless of age. Furthermore, the age distribution of individuals in skeletal collections may not align with that of individuals encountered in forensic casework, where younger adults are more prevalent. Therefore, the present study aims to explore the influence of age on the expression of the Walker [3] cranial traits and its impact on sex estimation accuracy in a South African sample. By exploring the relationship between age and sex estimation accuracies, this research seeks to enhance our understanding of the effects of age on the reliability of forensic anthropological sex estimation methods.

## Materials and methods

### Sample

The sample comprised of the skulls of 453 black, white, and coloured South African males and females from the Pretoria Bone Collection (University of Pretoria) and the Raymond A. Dart Collection of Modern Human Skeletons (University of the Witwatersrand) in South Africa. Ethical approval was obtained to conduct this research (Reference numbers 588/2022 and DMC2018). The Pretoria Bone Collection and the Raymond A. Dart Collection represent the two largest documented skeletal assemblages in South Africa. Both repositories are cadaver-derived, with recent skeletal material obtained from body donor programs managed by the respective medical schools [25, 26]. Although the Pretoria Bone Collection (1943-Present) is more recent in origin compared to the Raymond A. Dart Collection (1921-Present), the individuals housed in both repositories originate primarily from the 20th century to the present day [25, 26]. The compositions of both collections (in terms of temporality as well as age, sex, and population affinity) are considered similar and representative of the same resource pool [28]. For the current study, Kruskal-Wallis tests were conducted to assess differences in trait scores between the samples from the two repositories. No statistically significant differences were found; therefore, the samples were pooled together for further analysis.

The cranium has previously been reported to reach its adult size and shape during adolescence, as young as 12 years of age [11;17]. A lower age limit of 14 years was thus selected to maximise the sample size; although only two individuals in the sample were younger than 18 years of age. No upper age limit was selected. Ultimately, the overall age range for the sample was between 14 and 108 years. The sample was then subdivided into ten age cohorts at 10-year increments (Table 1). As this study made use of convenience sampling, the number of individuals per cohort, as well as the demographic distribution within each cohort varies. As a result, the youngest and oldest cohorts consist of very few individuals; thus, sample size should be taken into consideration when interpreting the results. Individuals with visible pathology, ante-mortem trauma, and/or post-mortem damage that affected the accurate scoring of more than one of the traits were excluded.

**Table 1:** Total number of individuals in each age cohort and the sex and population affinity distribution of the sample

	Sex		Population affinity			Total
Age Cohort	Females	Males	BSA^1^	WSA^2^	CSA^3^	*n*
< 20	5	4	6	1	2	9
20–29	30	30	53	2	5	60
30–39	30	30	57	0	3	60
40–49	30	31	54	6	1	61
50–59	30	31	50	10	1	61
60–69	30	30	40	19	1	60
70–79	30	30	36	24	0	60
80–89	30	30	17	43	0	60
90–99	9	11	4	16	0	20
> 99	0	2	1	0	1	2
TOTAL	**224**	**229**	**318**	**121**	**14**	**453**

^1^BSA: Black South African, ^2^WSA: White South African, ^3^CSA: Coloured South African

**Fig. 1:**
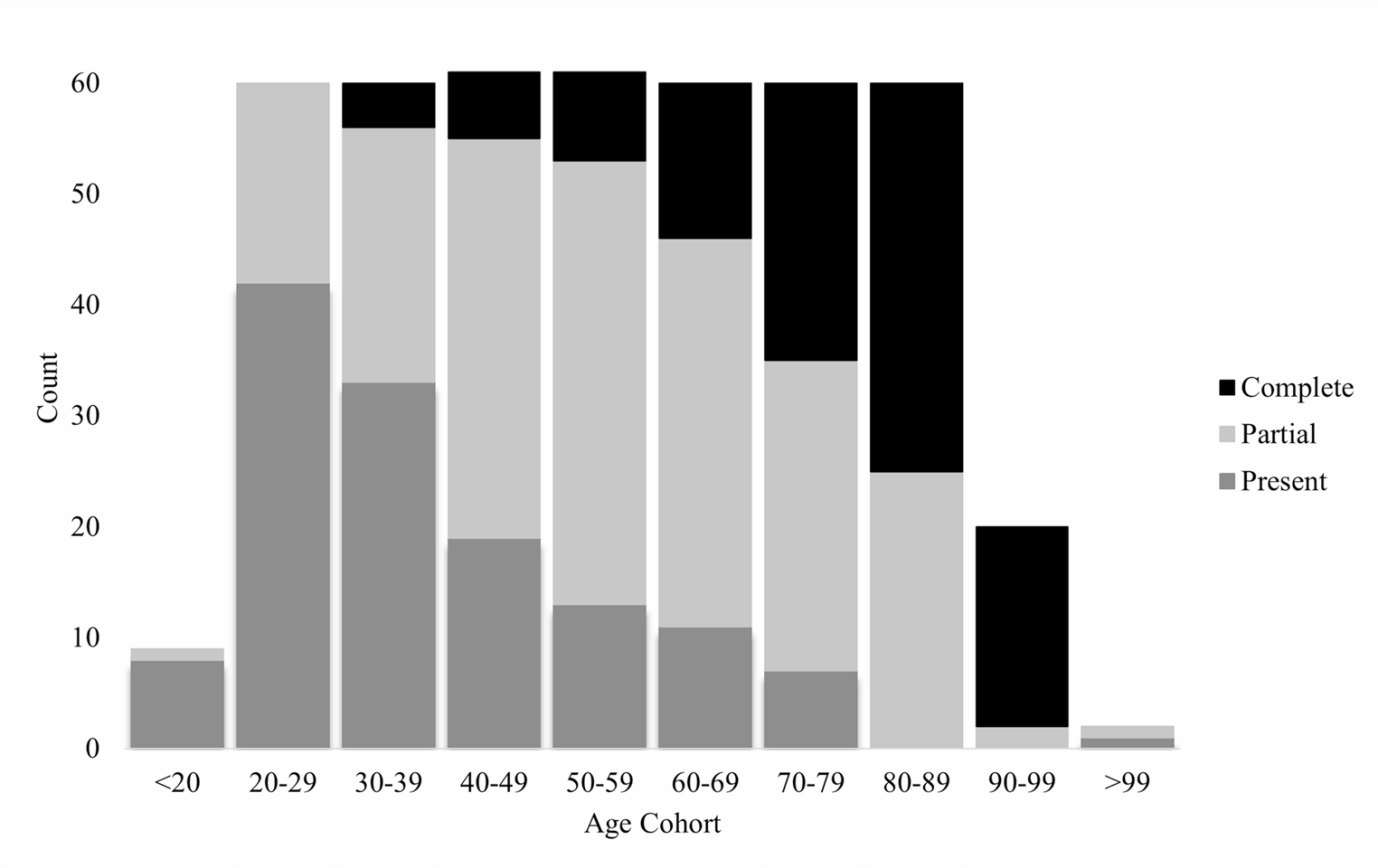
Frequency of individuals in the sample with present and complete dentition, partial edentulism and complete edentulism

Individuals with partial or complete edentulism were included in the sample as a large number of individuals in skeletal collections (and in the South African population) demonstrate edentulism to some degree. Partial edentulism can be defined as the antemortem loss of one or more teeth (excluding M3s) in one quadrant whereas complete edentulism is defined as the complete loss of teeth antemortem. The frequency of edentulism (partial and complete) for each age cohort of the sample is represented graphically in Fig. 1.

The frequency distribution shows that the percentage of individuals with edentulism increases with age, with the greatest percentage of individuals with edentulism concentrated in the older cohorts. Furthermore, partial edentulism is shown to be more concentrated in the middle-aged cohorts (40–69 years). To determine whether individuals who exhibited edentulism could be included in the study, a preliminary correlation between edentulism and the traits was conducted. A Spearman’s correlation was conducted to assess the association between edentulism and each of the traits prior to pooling the sample. The traits demonstrated very weak correlations to edentulism; The nuchal crest was the only trait with significant, but weak, correlation with tooth loss (*r* = 0.122; *p* = 0.001**)**. This supports the inclusion of edentulous individuals in the sample.

### Method

The five Walker [3] traits were visually assessed and scored for each skull in the sample (Table 2). Each trait was scored on an ordinal scale ranging between 1 and 5 using the definitions and line drawings as described in the methods of the original paper [3]. In the case of bilateral traits, only the left side was scored.

**Table 2:** List of the Walker [3] traits on the skull and their abbreviations

	Traits	Abbreviation	Scoring*
1	Glabella	Gla	1–5
2	Mastoid Process	Ma	1–5
3	Mental Eminence	Me	1–5
4	Nuchal Crest	Nu	1–5
5	Supra-orbital Margin	Or	1–5

*A score of 1 demonstrates maximum feminine expression, and a score of 5 maximum masculine expression.

### Statistical analyses

All statistical analyses were completed with R (version 4.0.5) and the RStudio environment (version v1.4.1106) [29].

Observer agreement was tested on 10 skulls (ranging in age from 16 to 69 years) randomly selected from the sample. These 10 skulls included a combination of black and white South African males and females to encompass a range of trait expressions. The first author (SKH) underwent a training period to become proficient with the method and rescored the skulls two weeks apart to gauge the intra-observer repeatability. For the inter-observer agreement, an additional observer (LL) with more than 10 years of experience with the method also scored the skulls. Since the Walker [3] traits are scored on an ordinal scale, a quadratic weighted Cohen’s Kappa was calculated for each trait using the *irr* package in R [30]. The calculated values were then interpreted using the descriptions by Landis and Koch [31] to show the strength of agreement between the scores.

Frequency distributions were calculated for each of the traits to analyse the distribution of scores within the sample. The frequency of each trait score was calculated for each trait separately subdivided into the different age cohorts. A Kruskal Wallis test was used to compare the scores for each trait among the different cohorts. A *post hoc* Dunn’s test was then calculated with a Bonferroni correction to further explore the results obtained with the Kruskal Wallis test. The Kruskal Wallis test is a non-parametric test suitable for ordinal data and is not subject to assumptions such as normality or homogeneity of variance. Results obtained with Kruskal Wallis are considered significant when *p* < 0.05. The Dunn’s test is required to determine which groups in a multiple comparison demonstrates significant differences. The Bonferroni correction counteracts the effects of multiple comparisons and prevents increased probability of Type I errors occurring [32]. Two different iterations were conducted: first the entire sample (sexes and population affinities) was pooled together; thereafter, the sample was separated according to sex to assess if there are different sex-specific trends among the age cohorts. The results of the Kruskal Wallis test were subsequently used *post hoc* to subdivide the sample for the classification models. A Kruskal Wallis test was also used to test for population differences in the sample.

Classification models were created to establish the accuracy of the traits when the sample is pooled together, and when the sample is divided according to the Kruskal Wallis results (i.e., age-specific standards). To achieve this, multiple classification methods were employed. The classification methods utilised a posterior probability threshold of 95.0% for reliable estimation of sex. Firstly, ordinal logistic regression (LR) was selected to test the sex estimation accuracy of the model as this is the method used in the original paper [3] as well as the South African-specific standards [4]. LR demonstrates the relationship between the traits and the probability of an individual classifying as male or female [4, 33]. The LR models were built using all traits simultaneously, with different weighted combinations assigned to the traits. Secondly, random forest modelling (RFMs) was selected as an additional method as it is becoming a more commonly employed classification technique in the field of forensic anthropology. As an example, RFM is used in MorphoPASSE, which is a computer program where trait scores from the skull and os coxa can be entered in a user interface for statistical analysis to produce sex estimates with associated probabilities for unknown individuals [34]. RFM is an ensemble machine-learning method that is capable of capturing complex, non-linear relationships between variables without the strict statistical assumptions associated with methods such as LR [33]. Couronné and colleagues [33] stated that LR allows the focus to be on both prediction and explanation whereas RFMs are more prediction focused. As LR is model based, the predictive performance is dependent on whether and how well the data followed the assumed model. RFMs utilise a multitude of decision trees that are built based on a bootstrap sample that is randomly drawn from the original dataset. Although RFM is often considered to be more complex to interpret and tune, it has been shown to outperform LR in both accuracy and flexibility [7, 33]. In the current study, the RFMs were set up so that 75% of the sample was used to train the model and the remaining 25% was used as a hold-out sample to test the model, providing an independent validation of the model performance. The *randomForest* package in R was used to conduct the RFM classifications [35]. The positive predictive performance was evaluated on three different cohort specific models (one pooled, one younger, and one older). In instances of missing values for a certain trait, the mode of the trait was used as a data imputation technique; the mode was calculated for the specific sex and population group that the individual belongs to. Data imputation was only conducted when less than 10% of the values for the trait in question were missing to limit the introduction of bias into the dataset and potentially masking any variation in the sample [36]. Finally, sex bias was calculated for the LR and RFMs by subtracting the female accuracy from the male accuracy. Sex bias indicates whether a certain sex group is classifying more accurately than the other and is useful for identifying trends in the scores that may lead to misclassification.

## Results

### Inter- and intra-observer agreement

Inter- and intra-observer agreement was tested to confirm the repeatability of the traits and accuracy of the study to ensure that observer differences could be excluded as a variable and all significant differences identified could be attributed to age-related changes. Table 3 presents the Kappa values. The intra-observer agreement ranged from “No agreement” (−0.13) to “Perfect agreement” (1.00), with the mental eminence and nuchal crest performing the worst and best, respectively. When comparing the results for the inter-observer agreement, the Kappa values were slightly lower, ranging from “Agreement equivalent to chance” (0.00) to “Near perfect” (0.87). Once again, the mental eminence performed the worst, and the nuchal crest performed the best. Despite attempts to improve the repeatability of the mental eminence prior to the main data collection, the observer agreement remained extremely low (as reported in Table 3). As such, the mental eminence was excluded from further analyses. This exclusion is also consistent with recommendations when using MorphoPASSE as the mental eminence is prone to poor repeatability [34].

**Table 3:** Quadratic-weighted Cohen’s Kappa and values for the inter- and intra-observer agreement with the associated description following Landis and Koch [31]

Trait	Intra-observer			Inter-observer	
	Kappa	Description		Kappa	Description
Gla	0.83	Near perfect		0.84	Near perfect
Ma	0.76	Substantial		0.77	Substantial
Or	0.68	Substantial		0.46	Moderate
Nu	1.00	Perfect		0.87	Near perfect
Me	−0.13	None		0.00	Equivalent to chance

### Exploratory analyses

**Fig. 2:**
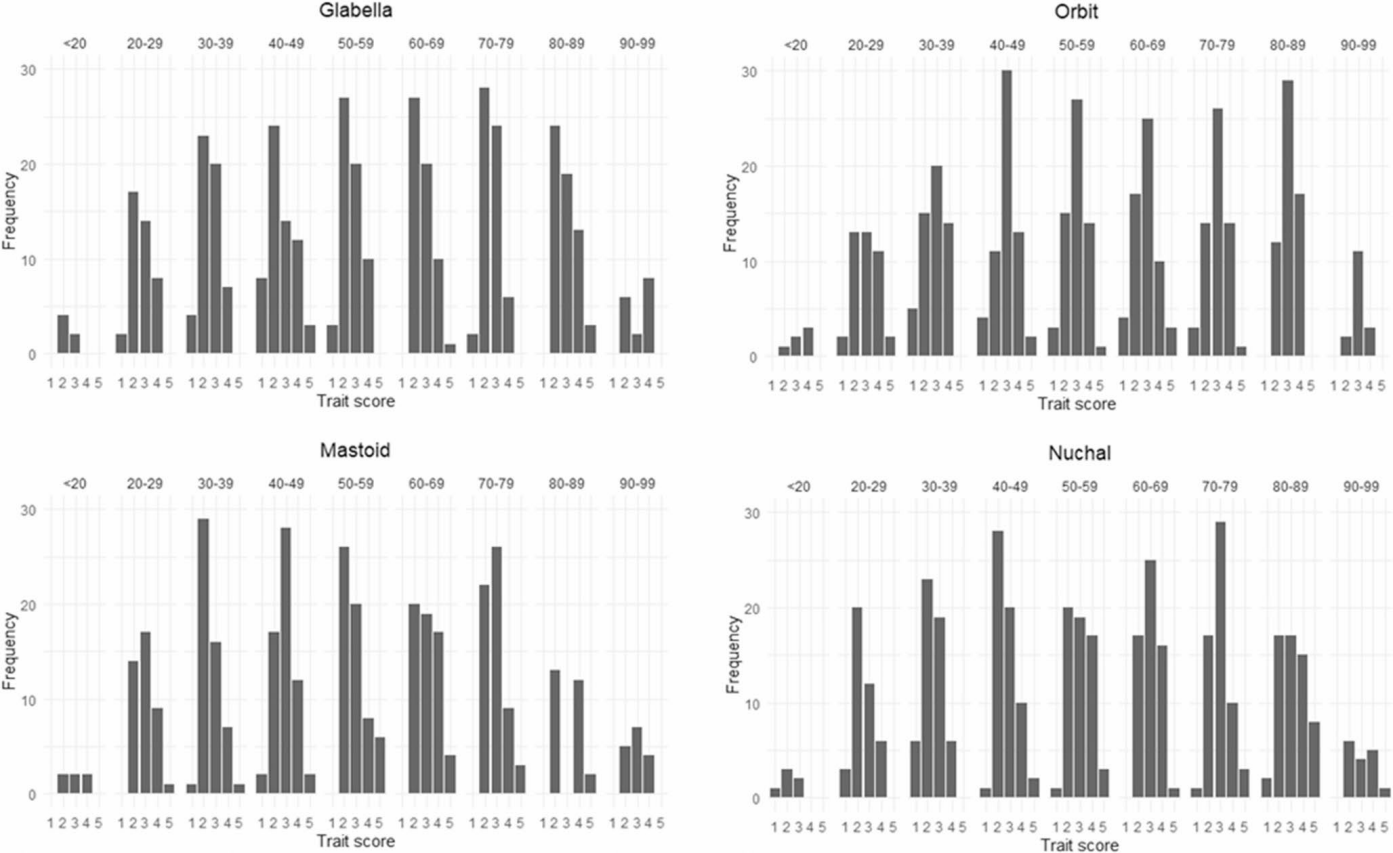
Frequency distribution of each trait by cohort (sexes pooled)

**Fig. 3:**
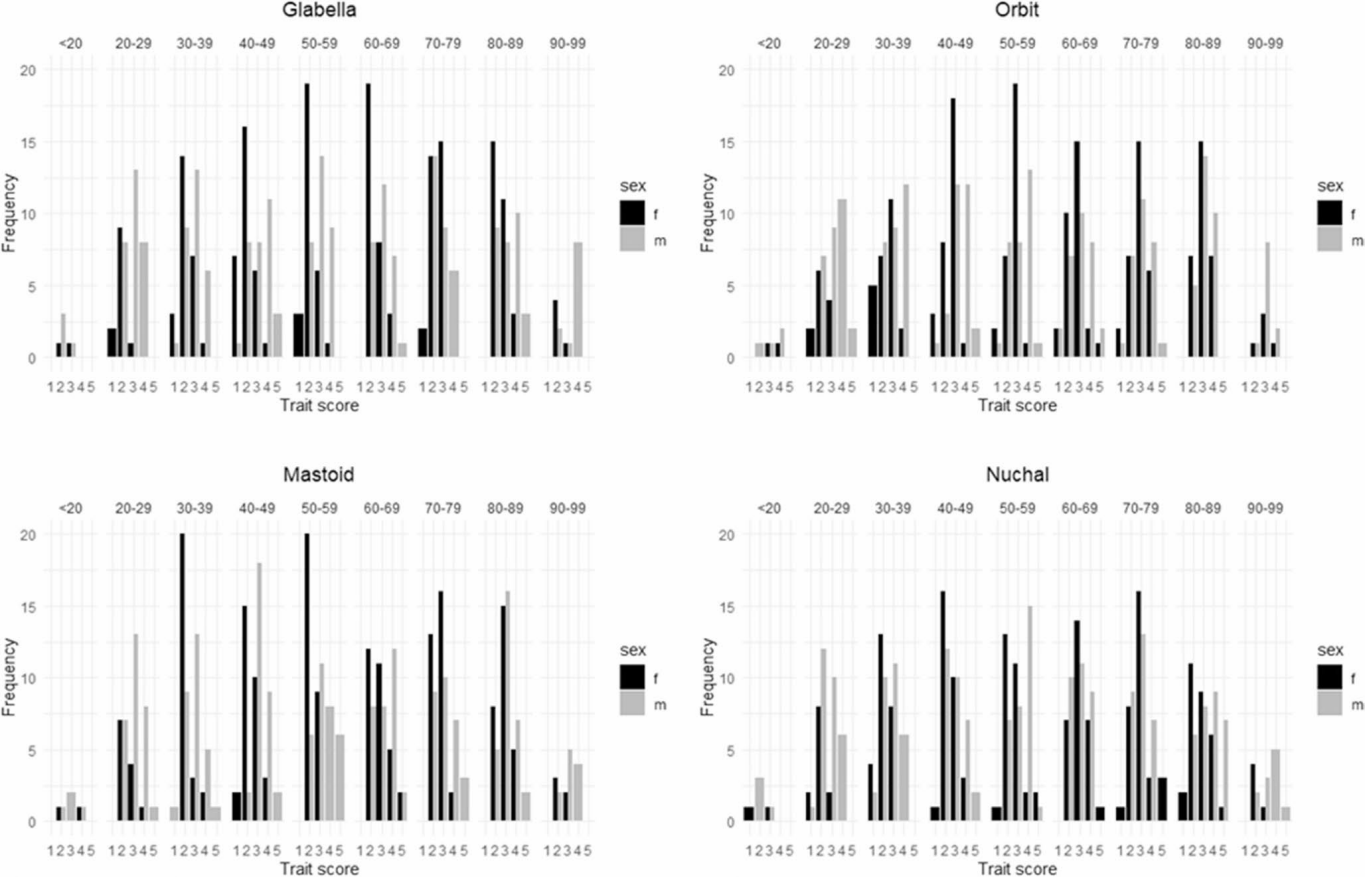
Frequency distribution of each trait by cohort (separated by sex)

The frequencies for each trait score were calculated per age cohort with the sexes and population groups pooled (Fig. 2), as well as separated by sex (Fig. 3). Please refer to the supplementary material for detailed tables presenting the trait frequencies. For the pooled sample, the frequency distribution shows that the greatest frequency of scores for all traits is concentrated around the intermediate scores (scores 2 to 4) with the older cohorts demonstrating a greater frequency for a score of 5 (2.64% vs. 0.95%) and the younger cohorts demonstrating a greater frequency for a score of 1 (6.51% vs. 2.25%) (Fig. 2). This trend was also observed when assessing the sexes separately. The female frequency distribution shows that the greatest frequency (92.38%) of scores is still concentrated around the intermediate scores (scores 2 to 4) for all of the traits. The older cohorts demonstrated a slightly greater frequency for a score of 4 or 5 (13.12% versus 11.20%), whereas the younger cohorts demonstrated a greater frequency for a score of 1 or 2 (59.67% versus 46.08%) (Fig. 3). Females younger than 40 years of age are more likely to be given a score of 2 for the glabella, mastoid process, nuchal crest, and mental eminence, whereas females older than 60 years of age are more likely to be given a score of 3 for the mastoid process, supra-orbital margin, nuchal crest, and the mental eminence. For the males, the younger cohorts once again displayed a score of a 1 or 2 more frequently that the older cohorts (individuals in the younger cohorts displayed a score of a 1 or 2 33.31% of the time, whereas the older cohorts scored a 1 or 2 only 20.36% of the time). Similarly, the older cohorts demonstrated a greater frequency for a score of 4 or 5 (40.83%) than the younger cohorts (26.93%) for all of the traits.

In terms of age, the results of the Kruskal Wallis test showed that significant differences existed only for the nuchal crest between some of the cohort groups (*p* < 0.05), where the older cohorts demonstrated a greater tendency to receive higher scores than the younger cohorts (Table 4). More specifically, the results indicate that significant differences exist between those younger than 40 years of age and those older than 40 years of age. In particular, the *post hoc* Dunn’s test showed that the younger cohorts (especially the 20–29-year cohort) differed the most from the older cohorts. Table 4 shows only the cohorts where significant differences were present; for a more detailed table representing all the age cohorts refer to the supplementary material (Table S1). None of the other traits demonstrated any significant differences when comparing the age cohorts with the sexes pooled. When the sample is further separated according to sex the Kruskal Wallis test showed that among the females significant differences exist between some of the cohort groups for both the nuchal crest and the supra-orbital margin. However, the differences observed for the supra-orbital margin were only significant for one cohort comparison (20–29 versus 80–89-year-olds). The Dunn’s test highlighted that the younger cohorts (especially the 20-29- and 30–39-year-olds) differed the most from the older cohorts (Table 5). No significant differences were present between any of the age cohort groups for any of the traits for the male sample. Thus, the most notable differences are observed among females.

**Table 4:** Significant differences observed among different age cohorts for the nuchal crest in a pooled sample

Cohort comparison	*P*-value
< 20| 80–89	**< 0.05**
20–29| 50–59	**< 0.05**
20–29| 60–69	**< 0.01**
20–29| 70–79	**< 0.05**
20–29| 80–89	**< 0.01**
30–39| 60–69	**< 0.05**
30–39| 80–89	**< 0.01**

**Table 5:** Significant differences present in females of different age cohorts using the Kruskal Wallis test with a post-hoc Dunn’s test

Trait	Cohort comparison	*P*-value
Or	20–29| 80–89	**< 0.05**
Nu	20–29| 60–69	**< 0.01**
	30–39| 60–69	**< 0.01**
	20–29| 70–79	**< 0.01**
	30–39| 70–79	**< 0.05**

It should be acknowledged that in terms of population affinity, significant differences were also observed for the nuchal crest with a Kruskal Wallis test (*p* < 0.05), while none of the other traits demonstrated a statistically significant association with population affinity. Closer scrutiny of the sample composition (as presented in Table 1) revealed a skewed population distribution among the cohorts, where the individuals younger than 40 years are primarily black South Africans, while the individuals older than 80 years are primarily white South Africans. Thus, the apparent significant age differences noted for the nuchal crest are most likely the result of population variation in the expression of the trait rather than true age variation in its expression. More specifically, among the females the older cohorts consisting primarily of white South African females, were assigned greater scores than the younger cohort consisting primarily of black South African females. 

### Classification models

Despite limited significant differences reported with the Kruskal Wallis tests, classification models were created to assess if separating the sample by age would influence the accuracy with which sex could be estimated using the traits. Based on the exploratory results, the sample was subdivided into two groups using 40 years as a sectioning point; thus, the younger subgroup included all individuals younger than 40 years (*n* = 129), and the older subgroup included all individuals older than 40 years (*n* = 324). Classification models were firstly created with the entire sample pooled, after which additional age-specific models were created for comparative purposes. Each model was assessed using both LR and RFM to explore the performance of the different statistical methods (Table 6).

**Table 6:** Classification accuracies (%) for the different models using LR and RFM

Model		Pooled sample	Younger sample	Older sample
LR	Combined	70.7	79.3	69.1
	Females	75.0	75.7	65.4
	Males	66.4	82.8	72.8
	Sex bias*	−8.6	7.1	7.4
RFMs	Combined	71.7	75.3	69.8
	Females	75.0	66.7	78.5
	Males	68.4	80.9	61.5
	Sex bias*	−6.6	14.2	−17.0

*Sex bias = Male percent correct – female percent correct x 100

Overall, the LR model for the pooled sample achieved a classification accuracy of 70.7%, with females generally classifying better than the males (75.0% and 66.4%, respectively). The glabella, mastoid process and supra-orbital margin were shown to be statistically significant and therefore have high variable importance for the LR function. The nuchal crest was not noted to be significant. When the sample is separated by age, the younger sample demonstrates a marked increase (79.3%), with males classifying correctly more frequently than females. The older sample demonstrated a similar overall accuracy compared to the pooled sample (69.1%); however, the female classification accuracy is lower than was observed with the pooled sample. The glabella, mastoid process and supra-orbital margin were shown to be statistically significant and have a high variable importance for the younger sample LR function, but for the older sample only the glabella and mastoid process were noted to be significant.

**Fig. 4:**
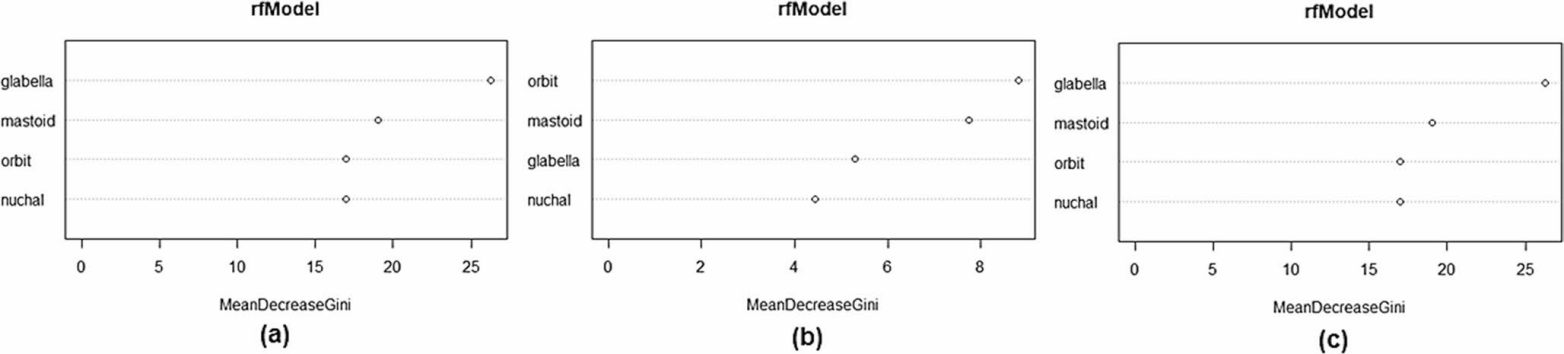
Comparison of RFM model variable importance for the (**a**) pooled sample, (**b**) younger sample, and (**c**) older sample

The RFM models yielded overall accuracies that were comparable to the LR models; however, the performance of the individual sexes showed different patterns of misclassification with regards to the older sample in the age-specific model. For the younger sample model, the females have a decreased accuracy compared to the pooled sample, while the males have a marked increase in accuracy which resulted in a large sex bias (14.2%). Conversely, within the older sample the female accuracy was markedly increased (78.5%) compared to the males (61.5%), once again resulting in a large sex bias (17.0%). In terms of variable importance (measured by the mean decrease in the Gini index), both the pooled sample and older sample models ranked the glabella as the most discriminatory variable, followed by the mastoid process, supra-orbital margin, and nuchal crest (in that order) (Fig. 4). For the younger sample the supra-orbital margin was considered most discriminatory, followed by the mastoid process, glabella, and the nuchal crest.

## Discussion

Previous research has ascribed misclassification in sex estimation using the Walker [3] traits as the result of changes that occur with advancing age [9, 11–17]. The current study explored potential trends in the trait scores that may be attributable to age and assessed how age can affect the positive predictive performance of the traits when estimating sex.

Overall, very few statistically significant differences were observed in the trait scores among the age cohorts. More specifically, the nuchal crest was the only trait to demonstrate any significant differences in the pooled sample, where individuals younger than forty years of age were noted to differ from individuals older than forty years of age. The nuchal crest is a major attachment site for structures of the head and neck, including the nuchal ligament, and the trapezius, semispinalis capitis, and splenius capitis muscles. De La Paz and colleagues [37] analysed the muscle structures and attachment sites of the head and neck for sex, population, and age-related differences. The study focused on the anatomical relation of the muscle and subsequent attachment sites to the morphology of the nuchal crest [37]. Typically, nuchal crests that are considered to be more robust (i.e., that would be given a score of 5) include both a prominent, often hooked nuchal crest paired with rough nuchal lines. In their results, De La Paz and colleagues [37] did not find significant sex or age-related differences in the muscle attachment site itself; however, the authors did identify variation in the types of attachments. The results demonstrated the variable attachment of the nuchal ligament and trapezius muscle, as a greater percentage of individuals exhibit no proximal cranial attachment by the superior portion of the trapezius muscle. Thus, the appearance of the nuchal crest as employed by forensic anthropologists may be influenced by the type of muscle attachment so that the nuchal area appears more robust. The current study also identified a significant, but weak, correlation between edentulism and the nuchal crest. The significant correlation between antemortem tooth loss and the nuchal crest may have been a factor in the significant differences identified between the age cohorts for the trait. Edentulism has a direct resultant effect on the masticatory function of the mandible and maxilla and therefore the muscles and skeletal structures related to this function and other functions of the head and neck may be affected [12, 18]. Factors that affect the muscle may directly affect the attachment site of the muscle to the bone and thus it is imperative that further studies are conducted to identify the effect of the correlation between edentulism and the nuchal crest as it was beyond the scope of the current study.

This apparent robusticity is not necessarily the result of sexual dimorphism and does not appear to be the result of aging. Indeed, the fact that the nuchal crest was noted to differ significantly between the population groups need to be considered. With the current sample having a skewed distribution with a greater proportion of young black South Africans compared to a greater proportion of older white South Africans, the apparent robusticity in the older cohorts is likely population variation. As is consistent with previous South African studies, white South Africans (both male and female) are on average more robust than black and coloured South Africans [4, 7], and this observation could explain why the nuchal crest of the older cohorts appear more robust in the current study. One key difference between the current findings with those in previous South African studies, is that Krüger and colleagues [4, 7] observed significant population differences for all traits rather than just the nuchal crest.

When the sample is divided so that the sexes are assessed separately the results indicated that the males did not demonstrate any significant differences for the nuchal crest, and all significant differences were observed in the female sample. More specifically, younger females (between 20 and 39 years) differed from the older females (between 60 and 79 years), while females that fell within the middle-aged cohort did not differ significantly from either the younger or older cohorts. Thus, the significant differences noted for the nuchal crest in the pooled sample was most likely due to significant differences between the cohorts of female individuals, and thereby emphasises the importance of exploring the sexes separately to more effectively identify age-related trends in craniofacial changes as each sex appears to be undergoing changes differently. Changes in the nuchal crest are assumed to be the result of continued and increasing strain of the nuchal ligament and trapezius muscle throughout an individual’s lifespan. Additionally, any stress experienced by the vertebral column as the result of morphological changes, such as vertebral wedging that may occur with osteoporosis or general vertebral body compression that occurs with age, would also have an effect on the nuchal ligament as it is a superior and posterior extension of the supraspinous ligament [4, 7, 38, 39].

Sex differences in musculoskeletal markers are typically attributed to differences in activity patterns or sexual division of labour, with males frequently engaging in harder physical labour resulting in increased hypertrophy of muscles and muscle attachment sites [40, 41] noted that both sex and age play a role in the robusticity of muscle markers of the humerus, where females demonstrated slighter entheseal changes compared to their male counterparts. Yet the results from the current study did not follow this trend with the nuchal crest. It should be acknowledged that the majority of studies that have explored musculoskeletal activity markers and age have looked at postcranial skeletal elements, and changes on muscle markers of the cranium are incompletely described in the literature. However, further factors that should be considered to better understand age-related changes to the nuchal crest (in addition to sexual dimorphism) include body size, muscle size, and muscle attachment size [37, 40, 41] also argues that aggregate muscle marker analyses (i.e., from more than one muscle attachment site) be conducted to gain a better understanding of the effects that the above factors have on the muscle attachments sites of individuals. Additionally, it should be acknowledged that despite the mastoid process also being a major muscle attachment site, this region demonstrated no significant associations with age or population affinity in the current study.

In addition to the nuchal crest, the females also demonstrated some significant differences (between the 20-year-old and 80-year-old cohorts) for the supra-orbital margin when assessing the sexes separately. Once again, the males did not present with any differences, nor were the differences observed with the pooled sample. Many other studies have analysed age changes to the orbit; however, these studies mainly focused on metric changes. Kahn and Shaw [42] noted significant changes with age to the orbital width and area in both males and females, and suggested that dramatic changes occur to the entire bony orbit throughout an individual’s life. Özer and colleagues [43] found slight correlations between changes in the width and height of the orbital aperture and increasing age in females but not in males. Additionally, Ugradar and Lambros [44] identified a relationship between increasing orbital volume and increasing age in females. However, as the current study only identifies significant differences between two of the cohorts there is a distinct possibility that it is an artefact from the sample. Therefore, it is imperative that further studies be conducted to better explore sex differences in the aging of the orbit, especially with regards to morphoscopic methods.

Another aspect that should be considered is secular trends. While it was not within the scope of the study to explore secular trends, it should be considered that secular changes in the cranium may affect the traits. Godde [45] studied individuals from the Hamann-Todd and William M. Bass Skeletal Collections and identified the presence of some secular trends in the cranial morphoscopic traits in North American males and females from 1849 to 1960. Jantz and Jantz [46] and Grine et al. [47] identified metric secular trends that affect the cranial morphology in 19th to 20th century North Americans and South Africans, respectively. Unfortunately, to date the majority of the literature focuses on metric changes and there has been little to no focus on whether (and to what extent) the morphoscopic traits are affected in this regard. Further research should be conducted to determine if secular trends are present in the morphoscopic traits and whether such trends are occurring in the South African population.

The results of the current study demonstrated only a few significant differences among the age cohorts; however, this does not negate the existence of age-related differences. It is likely that the morphoscopic methods may be unable to effectively quantify the small differences caused by age-related changes between the cohorts. For example, it is unlikely that a female’s score would change so drastically (such as from a score of 1 to a score of 3) in one lifespan. The shift from a score of a very gracile 1 to a score of an intermediate 3 would be radical and would require a large amount stress and/or remodelling for the bone to alter so significantly. As was noted in previous literature that was carried out on different populations [9, 11–16], significant differences were present between the age cohorts, but were slight and did not have a considerable effect on the sex estimate (combined accuracy of 71.90%; <40 cohort accuracy of 69.10%; and > 40 cohort accuracy of 81.30%) [3, 16]. Thus, to account for the more subtle changes, metric methods and geometric morphometrics may be better able to quantify the more nuanced changes to the craniofacial skeleton that occur due to the aging process.

Despite limited significant differences, the sample was subdivided into two broad groups (younger versus older) to see if there would be any difference in classification accuracy that could possibly justify prior knowledge of age to estimate sex. The results from the classification models indicated a slight increase in accuracy in the younger sample, which showed that the younger age cohorts classify more accurately when separated from the older age cohorts. The increase in accuracy in the younger sample is largely due to a substantial increase in the accuracy of the younger males, with the younger females only showing a slight improvement. When the age cohort was preselected in the LR model, the accuracy of the younger males substantially increased which is most likely due to the removal of the more “masculine-presenting” older females. Interestingly, the preselection of age in the LR model resulted in the inversion of the sex bias indicating that the males (in both the younger and older sample) classify better when the respective younger/older females are not included in the model. Similarly, the preselection of age in the RFM resulted in a substantial increase in the accuracy of the younger males. Contrastingly, the RFM age-preselection resulted in a decrease in the accuracy of the older sample males and a slight increase in accuracy of the older sample females. The RFMs more clearly demonstrate that the individual accuracies improve when the younger more “feminine-presenting” males are separated from the older more “masculine-presenting” females. However, the overall overlap in trait appearance for the more “feminine-presenting” younger group and the more “masculine-presenting” older group results in decreased RFM accuracies for the younger females and older males. It is essential to note that the differences between the models and related predictive performance may be due to model/sample limitations. Nevertheless, these results are consistent with the literature that states that females become more robust with age [23] resulting in a decrease in sexual dimorphism where their traits appear more masculine. However, the changes are slight and given the amount of overlap that naturally occurs with the traits, the accuracy is not greatly affected when age-specific standards are used.

Krogman and İscan [24] stated that, due to the effects of age on the skeleton, sex should only be estimated from the cranium for individuals between the ages of twenty and fifty-five years due to the increasing robusticity of females in older age cohorts. However, the classification accuracy observed with the older cohort classification model contests this, as the accuracy was not substantially lower than either the pooled or younger models. Thus, morphoscopic sex estimation can be performed with high accuracy in the South African population regardless of age. This supports other studies, such as that completed by Garvin and colleagues [10] and the study by Lesciotto and Doershuk [16], which stated that even though they found significant associations between age and the Walker [3] traits, the correlation was weak and did not influence the accuracy of the sex estimate or the traits enough to validate the creation of new standards for medicolegal contexts.

Finally, when assessing the performance of the two statistical methods—LR and RFM—both methods yielded similar overall accuracies for the pooled sample. However, closer scrutiny revealed that RFM exhibited a much greater sex bias with the age-specific samples. In the younger sample, males performed much better than females, while the older sample demonstrated the opposite trend, resulting in significant sex bias. Sex bias can introduce systematic error, where one sex is overestimated, which is undesirable for classification models. One potential reason for this is the weighting of traits and variable importance. The younger model ranked the supra-orbital margin as the most discriminatory variable, while the older model and pooled model placed more importance on the glabella. This similarity between the pooled and older sample results could be due to the older age group constituting majority of the pooled sample (*N* = 324/453), masking the contribution of the younger individuals in the pooled sample. Although previous studies have reported higher accuracies for RFM over LR [7], the findings of the current study show that the performance and choice of model depend heavily on dataset characteristics. When dividing the sample into age-specific subgroups, an imbalance in factors such as sample size and population affinity likely affected model performance and led to overfitting [34]. Therefore, it is crucial to carefully select samples to avoid overfitting and ensure optimal performance. The results also indicate that overall classification accuracy is not always the best measure of model performance. Other metrics, such as sex bias, sensitivity, specificity, and probability, should also be considered when developing classification models and standards.

## Conclusion

The findings of this study indicate that the nuchal crest was the only trait to show significant differences among age cohorts, particularly among females in the sample. However, given the sample distribution, it should be acknowledged that population affinity likely influenced this significant result. The pre-selection of age prior to sex estimation did not substantially affect the classification outcomes. The observed significant differences may not solely be attributed to age-related changes, as factors such as population-specific differences, edentulism, secular trends, and the quasi-continuous nature of morphoscopic methods may have influenced the results. In age-specific models, the older sample exhibited a decrease in accuracy, while the accuracy improved when younger individuals were assessed separately. However, this increase was not substantial enough to justify the pre-selection of age or the isolation of younger age cohorts. Furthermore, dividing the sample into age-specific subsamples led to increased sex bias, particularly for the RFM models. Therefore, pre-selecting age before estimating sex using the Walker traits is not necessary for skeletal analysis in contemporary South African populations. However, the use of population-specific standards remains essential for more accurate classification.

## Supplementary Information

Below is the link to the electronic supplementary material.

Supplementary Material 1

## Data Availability

The datasets generated during and/or analysed during the current study are available from the corresponding author on reasonable request.
