# Supplementary material for: The impact of age-related changes in the skull on sex estimation using morphoscopic traits
Source: Int J Legal Med. 2025 Aug 6;139(6):2991–3003. doi: 10.1007/s00414-025-03568-1 (PMC12532765; doi:10.1007/s00414-025-03568-1)
Supplement: Supplementary file 1 — Supplementary Material 1 [file 414_2025_3568_MOESM1_ESM.docx]

| **Table S1.** Significant differences present between the nuchal crest of different age cohorts using the Kruskal Wallis test with a post hoc Dunn’s test and a Bonferroni correction. Bold indicates significant differences. | |
| --- | --- |
| **Cohort comparison** | **p-value** |
| <20 - 20-29 | 1.00 |
| <20 - 30-39 | 1.00 |
| <20 - 40-49 | 1.00 |
| <20 - 50-59 | 0.12 |
| <20 - 60-69 | 0.09 |
| <20 - 70-79 | 0.19 |
| <20 - 80-89 | **<0.05** |
| <20 - 90-99 | 0.27 |
| <20 - 99< | 1.00 |
| 20-29 - 30-39 | 1.00 |
| 20-29 - 40-49 | 1.00 |
| 20-29 - 50-59 | **<0.05** |
| 20-29 - 60-69 | **<0.01** |
| 20-29 - 70-79 | **<0.05** |
| 20-29 - 80-89 | **<0.01** |
| 20-29 - 90-99 | 0.40 |
| 20-29 - 99< | 1.00 |
| 30-39 - 40-49 | 1.00 |
| 30-39 - 50-59 | 0.07 |
| 30-39 - 60-69 | **<0.05** |
| 30-39 - 70-79 | 0.18 |
| 30-39 - 80-89 | **<0.01** |
| 30-39 - 90-99 | 0.86 |
| 30-39 - 99< | 1.00 |
| 40-49 - 50-59 | 1.00 |
| 40-49 - 60-69 | 1.00 |
| 40-49 - 70-79 | 1.00 |
| 40-49 - 80-89 | 0.87 |
| 40-49 - 90-99 | 1.00 |
| **Table S1. (continued)** | |
| **Cohort comparison** | **p-value** |
| 40-49 - 99< | 1.00 |
| 50-59 - 60-69 | 1.00 |
| 50-59 - 70-79 | 1.00 |
| 50-59 - 80-89 | 1.00 |
| 50-59 - 90-99 | 1.00 |
| 50-59 - 99< | 1.00 |
| 60-69 - 70-79 | 1.00 |
| 60-69 - 80-89 | 1.00 |
| 60-69 - 90-99 | 1.00 |
| 60-69 - 99< | 1.00 |
| 70-79 - 80-89 | 1.00 |
| 70-79 - 90-99 | 1.00 |
| 70-79 - 99< | 1.00 |
| 80-89 - 90-99 | 1.00 |
| 80-89 - 99< | 1.00 |
| 90-99 - 99< | 1.00 |

| **Exploratory analyses: Pooled sample**  The frequency percentage of a score was calculated by adding all the percentages up and dividing by the number of age cohorts multiplied by the number of traits. For example, the frequency percentage of a score of 5 for the younger age cohort was calculated as follows:  Frequency of score 5 for younger age cohort = [(Gla <20 score 5 %) + (Ma <20 score 5 %) + (Or <20 score 5 %) + (Nu <20 score 5 %) + (Me <20 score 5 %) + (Gla 20-29 score 5 %) + (Ma 20-29 score 5 %) + (Or 20-29 score 5 %) + (Nu 20-29 score 5 %) + (Me 20-29 score 5 %) + (Gla 30-39 score 5 %) + (Ma 30-39 score 5 %) + (Or 30-39 score 5 %) + (Nu 30-39 score 5 %) + (Me 30-39 score 5 %)] ÷ (3 x 5)  Frequency of score 5 for younger age cohort = [0.0 + 0.0 + 0.0 + 0.0 + 0.0 + 0.0 + 5.0 + 3.4 + 0.0 + 2.0 + 0.0 + 1.7 + 0.0 + 0.0 + 2.2]÷ (3 x 5)  Frequency of score 5 for younger age cohort = 0.95% | | | | | | | | | | | | | | | | | | | | |
| --- | --- | --- | --- | --- | --- | --- | --- | --- | --- | --- | --- | --- | --- | --- | --- | --- | --- | --- | --- | --- |
|  | | | | | | | | | | | | | | | | | | | | |
| **Table S2.** Trait frequencies for each age cohort in the pooled sample. | | | | | | | | | | | | | | | | | | | | |
|  | **Age Cohort** | | | | | | | | | | | | | | | | | | | |
|  | **<20** | | **20-29** | | **30-39** | | **40-49** | | **50-59** | | **60-69** | | **70-79** | | **80-89** | | **90-99** | | **99<** | |
| **Trait Scores** | **n** | **%** | **n** | **%** | **n** | **%** | **n** | **%** | **n** | **%** | **n** | **%** | **n** | **%** | **n** | **%** | **n** | **%** | **n** | **%** |
| **Gla** | (n = 9) | | (n = 60) | | (n = 60) | | (n = 61) | | (n = 61) | | (n = 58) | | (n = 60) | | (n = 60) | | (n = 20) | | (n = 2) | |
| 1 | 1 | 11.1 | 6 | 10.0 | 5 | 8.3 | 8 | 13.1 | 3 | 4.9 | 0 | 0.0 | 2 | 3.3 | 0 | 0.0 | 1 | 5.0 | 0 | 0.0 |
| 2 | 6 | 66.7 | 26 | 43.3 | 26 | 43.3 | 24 | 39.3 | 28 | 45.9 | 27 | 46.6 | 28 | 46.7 | 24 | 40.0 | 7 | 35.0 | 0 | 0.0 |
| 3 | 2 | 22.2 | 18 | 30.0 | 21 | 35.0 | 14 | 23.00 | 20 | 32.8 | 20 | 34.5 | 24 | 40.0 | 19 | 31.7 | 3 | 15.0 | 1 | 50.0 |
| 4 | 0 | 0.0 | 10 | 16.7 | 8 | 13.3 | 12 | 19.7 | 10 | 16.4 | 10 | 17.2 | 6 | 10.0 | 14 | 23.3 | 9 | 45.0 | 1 | 50.0 |
| 5 | 0 | 0.0 | 0 | 0.0 | 0 | 0.0 | 3 | 4.9 | 0 | 0.0 | 1 | 1.7 | 0 | 0.0 | 3 | 5.0 | 0 | 0.0 | 0 | 0.0 |
| **Ma** | (n = 9) | | (n = 60) | | (n = 60) | | (n = 61) | | (n = 61) | | (n = 60) | | (n = 60) | | (n = 59) | | (n = 20) | | (n = 2) | |
| 1 | 0 | 0.0 | 0 | 0.0 | 1 | 1.7 | 2 | 3.3 | 0 | 0.0 | 0 | 0.0 | 0 | 0.0 | 0 | 0.0 | 0 | 0.0 | 0 | 0.0 |
| 2 | 3 | 33.3 | 26 | 43.3 | 30 | 50.0 | 17 | 27.9 | 26 | 42.6 | 20 | 33.3 | 22 | 36.7 | 13 | 22.0 | 6 | 30.0 | 0 | 0.0 |
| 3 | 3 | 33.3 | 21 | 35.0 | 17 | 28.3 | 28 | 45.9 | 21 | 34.4 | 19 | 31.7 | 26 | 43.3 | 31 | 52.5 | 10 | 50.0 | 1 | 50.0 |
| 4 | 3 | 33.3 | 10 | 16.7 | 11 | 18.3 | 12 | 19.7 | 8 | 13.1 | 17 | 28.3 | 9 | 15.0 | 13 | 22.0 | 4 | 20.0 | 1 | 50.0 |
| 5 | 0 | 0.0 | 3 | 5.0 | 1 | 1.7 | 2 | 3.3 | 6 | 4.9 | 4 | 6.7 | 3 | 5.0 | 2 | 3.4 | 0 | 0.0 | 0 | 0.0 |
| **Or** | (n = 9) | | (n = 59) | | (n = 60) | | (n = 60) | | (n = 61) | | (n = 59) | | (n = 58) | | (n = 59) | | (n = 20) | | (n = 2) | |
| 1 | 0 | 0.0 | 6 | 10.2 | 5 | 8.3 | 4 | 6.7 | 3 | 4.9 | 4 | 6.8 | 3 | 5.2 | 0 | 0.0 | 0 | 0.0 | 0 | 0.0 |
| 2 | 2 | 22.2 | 17 | 28.8 | 19 | 31.7 | 11 | 18.3 | 16 | 26.2 | 17 | 28.8 | 14 | 24.1 | 12 | 20.3 | 3 | 15.0 | 0 | 0.0 |
| 3 | 4 | 44.4 | 21 | 35.6 | 21 | 35.0 | 30 | 50.0 | 27 | 44.3 | 25 | 42.4 | 26 | 44.8 | 29 | 49.2 | 13 | 65.0 | 1 | 50.0 |
| 4 | 3 | 33.3 | 13 | 22.0 | 15 | 25.0 | 13 | 21.7 | 14 | 23.0 | 10 | 17.0 | 14 | 24.1 | 18 | 30.5 | 4 | 20.0 | 1 | 50.0 |
| 5 | 0 | 0.0 | 2 | 3.4 | 0 | 0.0 | 2 | 3.3 | 1 | 1.6 | 3 | 5.1 | 1 | 1.7 | 0 | 0.0 | 0 | 0.0 | 0 | 0.0 |
|  |  | |  | |  | |  | |  | |  | |  | |  | |  | |  | |
|  |  | |  | |  | |  | |  | |  | |  | |  | |  | |  | |
|  |  | |  | |  | |  | |  | |  | |  | |  | |  | |  | |
|  |  | |  | |  | |  | |  | |  | |  | |  | |  | |  | |
| **Table S2. (continued)** | | | | | | | | | | | | | | | | | | | | |
|  | **Age Cohort** | | | | | | | | | | | | | | | | | | | |
|  | **<20** | | **20-29** | | **30-39** | | **40-49** | | **50-59** | | **60-69** | | **70-79** | | **80-89** | | **90-99** | | **99<** | |
| **Trait Scores** | **n** | **%** | **n** | **%** | **n** | **%** | **n** | **%** | **n** | **%** | **n** | **%** | **n** | **%** | **n** | **%** | **n** | **%** | **n** | **%** |
| **Nu** | (n = 9) | | (n = 60) | | (n = 60) | | (n = 61) | | (n = 61) | | (n = 59) | | (n = 60) | | (n = 60) | | (n = 20) | | (n = 2) | |
| 1 | 2 | 22.2 | 6 | 10.0 | 7 | 11.7 | 1 | 1.6 | 1 | 1.6 | 0 | 0.0 | 1 | 1.7 | 2 | 3.3 | 0 | 0.0 | 0 | 0.0 |
| 2 | 5 | 55.6 | 30 | 50.0 | 26 | 43.3 | 28 | 45.9 | 20 | 32.8 | 17 | 28.8 | 17 | 28.3 | 17 | 28.3 | 7 | 35.0 | 0 | 0.0 |
| 3 | 2 | 22.2 | 18 | 30.0 | 20 | 33.3 | 20 | 32.8 | 20 | 32.8 | 25 | 42.4 | 29 | 48.3 | 18 | 30.0 | 6 | 30.0 | 1 | 50.0 |
| 4 | 0 | 0.0 | 6 | 10.0 | 7 | 11.7 | 10 | 16.4 | 17 | 27.9 | 16 | 27.1 | 10 | 16.7 | 15 | 25.0 | 6 | 30.0 | 1 | 50.0 |
| 5 | 0 | 0.0 | 0 | 0.0 | 0 | 0.0 | 2 | 3.3 | 3 | 4.9 | 1 | 1.7 | 3 | 5.0 | 8 | 13.3 | 1 | 5.0 | 0 | 0.0 |
| **Me** | (n = 9) | | (n = 51) | | (n = 45) | | (n = 42) | | (n = 41) | | (n = 34) | | (n = 24) | | (n = 21) | | (n = 2) | | (n = 2) | |
| 1 | 0 | 0.0 | 1 | 2.0 | 1 | 2.2 | 1 | 2.4 | 1 | 2.4 | 1 | 2.9 | 0 | 0.0 | 2 | 9.5 | 0 | 0.0 | 0 | 0.0 |
| 2 | 1 | 11.1 | 19 | 37.3 | 19 | 42.2 | 10 | 23.8 | 8 | 19.5 | 6 | 17.7 | 7 | 29.2 | 4 | 19.1 | 1 | 50.0 | 1 | 50.0 |
| 3 | 3 | 33.3 | 23 | 45.1 | 18 | 40.0 | 15 | 35.7 | 16 | 39.0 | 9 | 26.5 | 6 | 25.0 | 6 | 28.6 | 1 | 50.0 | 1 | 50.0 |
| 4 | 3 | 33.3 | 7 | 13.7 | 6 | 13.3 | 15 | 35.7 | 15 | 36.6 | 17 | 50.0 | 11 | 45.8 | 8 | 38.1 | 0 | 0.0 | 0 | 0.0 |
| 5 | 0 | 0.0 | 1 | 2.0 | 1 | 2.2 | 1 | 2.4 | 1 | 2.4 | 1 | 2.9 | 0 | 0.0 | 1 | 4.8 | 0 | 0.0 | 0 | 0.0 |

| **Table S3.** Trait frequencies for each age cohort for females. | | | | | | | | | | | | | | | | | | | | |
| --- | --- | --- | --- | --- | --- | --- | --- | --- | --- | --- | --- | --- | --- | --- | --- | --- | --- | --- | --- | --- |
|  | **Age Cohort** | | | | | | | | | | | | | | | | | | | |
|  | **<20** | | **20-29** | | **30-39** | | **40-49** | | **50-59** | | **60-69** | | **70-79** | | **80-89** | | **90-99** | | **99<** | |
| **Trait Scores** | **n** | **%** | **n** | **%** | **n** | **%** | **n** | **%** | **n** | **%** | **n** | **%** | **n** | **%** | **n** | **%** | **n** | **%** | **n** | **%** |
| **Gla** | (n = 5) | | (n = 30) | | (n = 30) | | (n = 30) | | (n = 30) | | (n =30) | | (n = 30) | | (n = 30) | | (n = 9) | | (n = 0) | |
| 1 | 1 | 20.0 | 6 | 20.0 | 4 | 13.3 | 7 | 23.3 | 3 | 10.0 | 0 | 0.0 | 2 | 6.7 | 0 | 0.0 | 1 | 11.1 | 0 | 0.0 |
| 2 | 3 | 60.0 | 18 | 60.0 | 16 | 53.3 | 16 | 53.3 | 20 | 66.7 | 19 | 63.3 | 13 | 43.3 | 15 | 50.0 | 5 | 55.6 | 0 | 0.0 |
| 3 | 1 | 20.0 | 5 | 16.7 | 8 | 26.7 | 6 | 20.0 | 6 | 20.0 | 8 | 26.7 | 15 | 50.0 | 11 | 36.7 | 2 | 22.2 | 0 | 0.0 |
| 4 | 0 | 0.0 | 1 | 3.3 | 2 | 6.7 | 1 | 3.3 | 1 | 3.3 | 3 | 10.0 | 0 | 0.0 | 4 | 13.3 | 1 | 11.1 | 0 | 0.0 |
| 5 | 0 | 0.0 | 0 | 0.0 | 0 | 0.0 | 0 | 0.0 | 0 | 0.0 | 0 | 0.0 | 0 | 0.0 | 0 | 0.0 | 0 | 0.0 | 0 | 0.0 |
| **Ma** | (n = 5) | | (n = 30) | | (n = 30) | | (n = 30) | | (n = 30) | | (n = 30) | | (n = 30) | | (n = 29) | | (n = 9) | | (n = 0) | |
| 1 | 0 | 0.0 | 0 | 0.0 | 0 | 0.0 | 2 | 6.7 | 0 | 0.0 | 0 | 0.0 | 0 | 0.0 | 0 | 0.0 | 0 | 0.0 | 0 | 0.0 |
| 2 | 2 | 40.0 | 19 | 63.3 | 21 | 70.0 | 15 | 50.0 | 20 | 66.7 | 12 | 40.0 | 13 | 43.3 | 8 | 27.6 | 4 | 44.4 | 0 | 0.0 |
| 3 | 1 | 20.0 | 8 | 26.7 | 4 | 13.3 | 10 | 33.3 | 10 | 33.3 | 11 | 36.7 | 15 | 50.0 | 15 | 51.7 | 5 | 55.6 | 0 | 0.0 |
| 4 | 2 | 40.0 | 2 | 6.7 | 5 | 16.7 | 3 | 10.0 | 0 | 0.0 | 5 | 16.7 | 2 | 6.7 | 6 | 20.7 | 0 | 0.0 | 0 | 0.0 |
| 5 | 0 | 0.0 | 1 | 3.3 | 0 | 0.0 | 0 | 0.0 | 0 | 0.0 | 2 | 6.7 | 0 | 0.0 | 0 | 0.0 | 0 | 0.0 | 0 | 0.0 |
| **Or** | (n = 5) | | (n = 29) | | (n = 30) | | (n = 30) | | (n = 30) | | (n = 30) | | (n = 29) | | (n = 30) | | (n = 9) | | (n = 0) | |
| 1 | 0 | 0.0 | 6 | 20.7 | 5 | 16.7 | 3 | 10.0 | 2 | 6.7 | 2 | 6.7 | 2 | 6.9 | 0 | 0.0 | 0 | 0.0 | 0 | 0.0 |
| 2 | 1 | 20.0 | 10 | 34.5 | 11 | 36.7 | 8 | 26.7 | 8 | 26.7 | 10 | 33.3 | 7 | 24.1 | 7 | 23.3 | 2 | 22.2 | 0 | 0.0 |
| 3 | 3 | 60.0 | 11 | 37.9 | 12 | 40.0 | 18 | 60.0 | 19 | 63.3 | 15 | 50.0 | 15 | 51.7 | 15 | 50.0 | 5 | 55.6 | 0 | 0.0 |
| 4 | 1 | 20.0 | 2 | 6.9 | 2 | 6.7 | 1 | 3.3 | 1 | 3.3 | 2 | 6.7 | 5 | 17.2 | 8 | 26.7 | 2 | 22.2 | 0 | 0.0 |
| 5 | 0 | 0.0 | 0 | 0.0 | 0 | 0.0 | 0 | 0.0 | 0 | 0.0 | 1 | 3.3 | 0 | 0.0 | 0 | 0.0 | 0 | 0.0 | 0 | 0.0 |
|  | | | | | | | | | | | | | | | | | | | | |
| **Table S3. (continued)** | | | | | | | | | | | | | | | | | | | | |
|  | **Age Cohort** | | | | | | | | | | | | | | | | | | | |
|  | **<20** | | **20-29** | | **30-39** | | **40-49** | | **50-59** | | **60-69** | | **70-79** | | **80-89** | | **90-99** | | **99<** | |
| **Trait Scores** | **n** | **%** | **n** | **%** | **n** | **%** | **n** | **%** | **n** | **%** | **n** | **%** | **n** | **%** | **n** | **%** | **n** | **%** | **n** | **%** |
| **Nu** | (n =5) | | (n = 30) | | (n = 30) | | (n = 30) | | (n = 30) | | (n = 29) | | (n = 30) | | (n = 30) | | (n = 9) | | (n = 0) | |
| 1 | 2 | 40.0 | 5 | 16.7 | 5 | 16.7 | 1 | 3.3 | 1 | 3.3 | 0 | 0.0 | 1 | 3.3 | 2 | 6.7 | 0 | 0.0 | 0 | 0.0 |
| 2 | 2 | 40.0 | 18 | 60.0 | 16 | 53.3 | 16 | 53.3 | 13 | 43.3 | 7 | 24.1 | 8 | 26.7 | 11 | 36.7 | 5 | 55.6 | 0 | 0.0 |
| 3 | 1 | 20.0 | 7 | 23.3 | 9 | 30.0 | 10 | 33.3 | 12 | 40.0 | 14 | 48.3 | 15 | 50.0 | 10 | 33.3 | 3 | 33.3 | 0 | 0.0 |
| 4 | 0 | 0.0 | 0 | 0.0 | 0 | 0.0 | 3 | 10.0 | 2 | 6.7 | 7 | 24.1 | 3 | 10.0 | 6 | 20.0 | 1 | 11.1 | 0 | 0.0 |
| 5 | 0 | 0.0 | 0 | 0.0 | 0 | 0.0 | 0 | 0.0 | 2 | 6.7 | 1 | 3.5 | 3 | 10.0 | 1 | 3.3 | 0 | 0.0 | 0 | 0.0 |
| **Me** | (n = 4) | | (n = 28) | | (n = 24) | | (n = 18) | | (n = 17) | | (n = 18) | | (n = 8) | | (n = 8) | | (n = 1) | | (n = 0) | |
| 1 | 0 | 0.0 | 1 | 3.6 | 1 | 4.2 | 0 | 0.0 | 1 | 5.9 | 1 | 5.6 | 0 | 0.0 | 1 | 12.5 | 0 | 0.0 | 0 | 0.0 |
| 2 | 1 | 25.0 | 16 | 57.1 | 12 | 50.0 | 5 | 27.8 | 6 | 35.3 | 5 | 27.8 | 3 | 37.5 | 2 | 25.0 | 1 | 100.0 | 0 | 0.0 |
| 3 | 1 | 25.0 | 10 | 35.7 | 10 | 41.7 | 10 | 55.6 | 7 | 41.2 | 4 | 22.2 | 3 | 37.5 | 5 | 62.5 | 0 | 0.0 | 0 | 0.0 |
| 4 | 2 | 50.0 | 1 | 3.6 | 1 | 4.2 | 3 | 16.7 | 3 | 17.7 | 7 | 38.9 | 2 | 25.0 | 0 | 0.0 | 0 | 0.0 | 0 | 0.0 |
| 5 | 0 | 0.0 | 0 | 0.0 | 0 | 0.0 | 0 | 0.0 | 0 | 0.0 | 1 | 5.6 | 0 | 0.0 | 0 | 0.0 | 0 | 0.0 | 0 | 0.0 |

| **Table S4.** Frequencies for each trait score in each age cohort for males. | | | | | | | | | | | | | | | | | | | | |
| --- | --- | --- | --- | --- | --- | --- | --- | --- | --- | --- | --- | --- | --- | --- | --- | --- | --- | --- | --- | --- |
|  | **Age Cohort** | | | | | | | | | | | | | | | | | | | |
|  | **<20** | | **20-29** | | **30-39** | | **40-49** | | **50-59** | | **60-69** | | **70-79** | | **80-89** | | **90-99** | | **99<** | |
| **Trait Scores** | **n** | **%** | **n** | **%** | **n** | **%** | **n** | **%** | **n** | **%** | **n** | **%** | **n** | **%** | **n** | **%** | **n** | **%** | **n** | **%** |
| **Gla** | (n = 4) | | (n = 30) | | (n = 30) | | (n = 30) | | (n = 30) | | (n =28) | | (n = 30) | | (n = 30) | | (n = 11) | | (n = 2) | |
| 1 | 0 | 0.0 | 0 | 0.0 | 1 | 3.3 | 1 | 3.3 | 0 | 0.0 | 0 | 0.0 | 0 | 0.0 | 0 | 0.0 | 0 | 0.0 | 0 | 0.0 |
| 2 | 3 | 75.0 | 8 | 26.7 | 10 | 33.3 | 7 | 23.3 | 7 | 23.3 | 8 | 28.6 | 15 | 50.0 | 9 | 30.0 | 2 | 18.2 | 0 | 0.0 |
| 3 | 1 | 25.0 | 13 | 43.3 | 13 | 43.3 | 8 | 26.7 | 14 | 46.7 | 12 | 42.9 | 9 | 30.0 | 8 | 26.7 | 1 | 9.1 | 1 | 50.0 |
| 4 | 0 | 0.0 | 9 | 30.0 | 6 | 20.0 | 11 | 36.7 | 9 | 30.0 | 7 | 25.0 | 6 | 20.0 | 10 | 33.3 | 8 | 72.7 | 1 | 50.0 |
| 5 | 0 | 0.0 | 0 | 0.0 | 0 | 0.0 | 3 | 10.0 | 0 | 0.0 | 1 | 3.6 | 0 | 0.0 | 3 | 10.0 | 0 | 0.0 | 0 | 0.0 |
| **Ma** | (n = 4) | | (n = 30) | | (n = 30) | | (n = 30) | | (n = 30) | | (n = 30) | | (n = 30) | | (n = 30) | | (n = 11) | | (n = 2) | |
| 1 | 0 | 0.0 | 0 | 0.0 | 1 | 3.3 | 0 | 0.0 | 0 | 0.0 | 0 | 0.0 | 0 | 0.0 | 0 | 0.0 | 0 | 0.0 | 0 | 0.0 |
| 2 | 1 | 25.0 | 7 | 23.3 | 9 | 30.0 | 2 | 6.7 | 5 | 16.7 | 8 | 26.7 | 9 | 30.0 | 5 | 16.7 | 2 | 18.2 | 0 | 0.0 |
| 3 | 2 | 50.0 | 13 | 43.3 | 13 | 43.3 | 17 | 56.7 | 11 | 36.7 | 8 | 26.7 | 11 | 36.7 | 16 | 53.3 | 5 | 45.5 | 1 | 50.0 |
| 4 | 1 | 25.0 | 8 | 26.7 | 6 | 20.0 | 9 | 30.0 | 8 | 26.7 | 12 | 40.0 | 7 | 23.3 | 7 | 23.3 | 4 | 36.4 | 1 | 50.0 |
| 5 | 0 | 0.0 | 2 | 6.7 | 1 | 3.3 | 2 | 6.7 | 6 | 20.0 | 2 | 6.7 | 3 | 10.0 | 2 | 6.7 | 0 | 0.0 | 0 | 0.0 |
| **Or** | (n = 4) | | (n = 30) | | (n = 30) | | (n = 30) | | (n = 30) | | (n = 29) | | (n = 29) | | (n = 29) | | (n = 11) | | (n = 2) | |
| 1 | 0 | 0.0 | 0 | 0.0 | 0 | 0.0 | 1 | 3.3 | 1 | 3.3 | 2 | 6.9 | 1 | 3.5 | 0 | 0.0 | 0 | 0.0 | 0 | 0.0 |
| 2 | 1 | 25.0 | 7 | 23.3 | 8 | 26.7 | 3 | 10.0 | 7 | 23.3 | 7 | 24.1 | 7 | 24.1 | 5 | 17.2 | 1 | 9.1 | 0 | 0.0 |
| 3 | 1 | 25.0 | 10 | 33.3 | 9 | 30.0 | 12 | 40.0 | 8 | 26.7 | 10 | 34.5 | 11 | 37.9 | 14 | 48.3 | 8 | 72.7 | 1 | 50.0 |
| 4 | 2 | 50.0 | 11 | 36.7 | 13 | 43.3 | 12 | 40.0 | 13 | 43.3 | 8 | 27.6 | 9 | 31.0 | 10 | 34.5 | 2 | 18.2 | 1 | 50.0 |
| 5 | 0 | 0.0 | 2 | 6.7 | 0 | 0.0 | 2 | 6.7 | 1 | 3.3 | 2 | 6.9 | 1 | 3.5 | 0 | 0.0 | 0 | 0.0 | 0 | 0.0 |
|  | | | | | | | | | | | | | | | | | | | | |
|  | | | | | | | | | | | | | | | | | | | | |
| **Table S4. (continued)** | | | | | | | | | | | | | | | | | | | | |
|  | **Age Cohort** | | | | | | | | | | | | | | | | | | | |
|  | **<20** | | **20-29** | | **30-39** | | **40-49** | | **50-59** | | **60-69** | | **70-79** | | **80-89** | | **90-99** | | **99<** | |
| **Trait Scores** | **n** | **%** | **n** | **%** | **n** | **%** | **n** | **%** | **n** | **%** | **n** | **%** | **n** | **%** | **n** | **%** | **n** | **%** | **n** | **%** |
| **Nu** | (n =4) | | (n = 30) | | (n = 30) | | (n = 30) | | (n = 30) | | (n = 30) | | (n = 30) | | (n = 30) | | (n = 11) | | (n = 2) | |
| 1 | 0 | 0.0 | 1 | 3.3 | 2 | 6.7 | 0 | 0.0 | 0 | 0.0 | 0 | 0.0 | 0 | 0.0 | 0 | 0.0 | 0 | 0.0 | 0 | 0.0 |
| 2 | 3 | 75.0 | 12 | 40.0 | 10 | 33.3 | 11 | 36.7 | 7 | 23.3 | 10 | 33.3 | 9 | 30.0 | 6 | 20.0 | 2 | 18.2 | 0 | 0.0 |
| 3 | 1 | 25.0 | 11 | 36.7 | 11 | 36.7 | 10 | 33.3 | 7 | 23.3 | 11 | 36.7 | 14 | 46.7 | 8 | 26.7 | 3 | 27.3 | 1 | 50.0 |
| 4 | 0 | 0.0 | 6 | 20.0 | 7 | 23.3 | 7 | 23.3 | 15 | 50.0 | 9 | 30.0 | 7 | 23.3 | 9 | 30.0 | 5 | 45.5 | 1 | 50.0 |
| 5 | 0 | 0.0 | 0 | 0.0 | 0 | 0.0 | 2 | 6.7 | 1 | 3.3 | 0 | 0.0 | 0 | 0.0 | 7 | 23.3 | 1 | 9.1 | 0 | 0.0 |
| **Me** | (n = 3) | | (n = 23) | | (n = 21) | | (n = 23) | | (n = 24) | | (n = 16) | | (n = 16) | | (n = 13) | | (n = 1) | | (n = 2) | |
| 1 | 0 | 0.0 | 0 | 0.0 | 0 | 0.0 | 1 | 4.4 | 0 | 0.0 | 0 | 0.0 | 0 | 0.0 | 1 | 7.7 | 0 | 0.0 | 0 | 0.0 |
| 2 | 0 | 0.0 | 3 | 13.0 | 7 | 33.3 | 4 | 17.4 | 2 | 8.3 | 1 | 6.3 | 4 | 25.0 | 2 | 15.4 | 0 | 0.0 | 1 | 50.0 |
| 3 | 2 | 66.7 | 13 | 56.5 | 8 | 38.1 | 5 | 21.7 | 9 | 37.5 | 5 | 31.3 | 3 | 18.8 | 1 | 7.7 | 1 | 50.0 | 1 | 50.0 |
| 4 | 1 | 33.3 | 6 | 26.1 | 5 | 23.8 | 12 | 52.2 | 12 | 50.0 | 10 | 62.5 | 9 | 56.3 | 8 | 61.5 | 0 | 0.0 | 0 | 0.0 |
| 5 | 0 | 0.0 | 1 | 4.4 | 1 | 4.8 | 1 | 4.4 | 1 | 4.2 | 0 | 0.0 | 0 | 0.0 | 1 | 7.7 | 0 | 0.0 | 0 | 0.0 |

|  |  |
| --- | --- |
|  |  |

**Supplementary Material Figure 1 –** Ridgeline plots illustrating the frequency distribution of each trait by cohort (sexes pooled)
